# Pre-clinical evaluation of antiproteases as potential candidates for HIV-1 pre-exposure prophylaxis

**DOI:** 10.3389/frph.2022.998913

**Published:** 2022-11-21

**Authors:** Carolina Herrera, Natalia Olejniczak, Laura Noël-Romas, Frank Plummer, Adam Burgener

**Affiliations:** ^1^ Immunology of Infection, Department of Infectious Disease, Faculty of Medicine, Imperial College London, London, United Kingdom; ^2^ Department of Pathology, Center for Global Health and Diseases, Case Western Reserve University, Cleveland, OH, United States; ^3^ Department of Obstetrics & Gynecology, University of Manitoba, Winnipeg, MB, Canada; ^4^ Department of Medical Microbiology and Infectious Diseases, University of Manitoba, Winnipeg, MB, Canada; ^5^ Department of Medicine Solna, Center for Molecular Medicine, Karolinska Institute, Karolinska University Hospital, Stockholm, Sweden

**Keywords:** HIV-1, antiproteases, ecto-cervix, pre-exposure prophylaxis, HESN, tissue explants

## Abstract

Previous studies on highly HIV-1-exposed, yet persistently seronegative women from the Punwami Sex Worker cohort in Kenya, have shed light on putative protective mechanisms, suggesting that mucosal immunological factors, such as antiproteases, could be mediating resistance to HIV-1 transmission in the female reproductive tract. Nine protease inhibitors were selected for this study: serpin B4, serpin A1, serpin A3, serpin C1, cystatin A, cystatin B, serpin B13, serpin B1 and α-2-macroglobulin-like-protein 1. We assessed in a pilot study, the activity of these antiproteases with cellular assays and an *ex vivo* HIV-1 challenge model of human ecto-cervical tissue explants. Preliminary findings with both models, cellular and tissue explants, established an order of inhibitory potency for the mucosal proteins as candidates for pre-exposure prophylaxis when mimicking pre-coital use. Combination of all antiproteases considered in this study was more active than any of the individual mucosal proteins. Furthermore, the migration of cells out of ecto-cervical explants was blocked indicating potential prevention of viral dissemination following amplification of the founder population. These findings constitute the base for further development of these mucosal protease inhibitors for prevention strategies.

## Introduction

Despite the progress in antiretroviral based HIV pre-exposure prophylaxis, there are still 1.5 million new HIV infections diagnosed per year. New infections disproportionately affect populations experiencing economic and gender inequities (1). The gender gap is most notable in areas of Sub-Saharan Africa, where more than half of those living with HIV and those newly infected with HIV are women (2). The majority of new HIV infections occur through mucosal transmission and with increasing prevalence of antiretroviral (ARV) drug resistance (3, 4), new pre-exposure prophylaxis (PrEP) strategies specifically designed to effectively protect the mucosal portals of viral entry and not solely ARV-based, need to be considered.

The Punwami Sex Worker cohort in Kenya includes women that have maintained high-risk sexual behavior and remained serologically and PCR negative for HIV (5, 6). Proteomic studies with cervicovaginal lavage (CVL) samples from this cohort suggest that mucosal immunological factors could be mediating resistance to HIV-1 transmission in the female reproductive tract. Antiproteases were among the proteins to be differentially expressed in CVL between HIV-1-resistant women and control groups (7, 8). The majority of over-abundant proteins were antiproteases, some with known anti-inflammatory and anti-HIV-1 activity. Serin protease inhibitors, known as serpins, play an important role in regulating inflammation and their absence can lead to severe inflammation, tissue damage, and disease (9, 10). Many serpins found overexpressed in the cohort of HIV-resistant women inhibit cathepsin G, which acts as a chemoattractant for macrophages and neutrophils (11, 12) and stimulates T cells (13); and elastase, which is known to increase the risk of HIV-1 infection and impair wound healing (14–17). Anti-inflammatory and anti-HIV-1 activity has also been described for cystatins and other protease inhibitory proteins such as α-2-macroglobulin-like-protein 1 (A2ML1) (18–23). In the present pilot study, we sought to evaluate the potential anti-HIV-1 activity of nine antiproteases including serpin B4, serpin A1, serpin A3, serpin C1, cystatin A, cystatin B, serpin B13, serpin B1 and A2ML1. We used cellular and mucosal tissue models to identify potential direct antiviral mechanisms or inhibition driven by anti-inflammatory-linked processes.

## Method

### Reagents and virus

Antiproteases (APs) were produced by GenScript (Piscataway, NJ, USA) *via* transient transfection of 293-6E cells with a recombinant plasmid encoding each AP. Purity was determined to be 80%–85% and functional analysis of the protein was confirmed using a Neutrophil Elastase Inhibitor Screening Kit (Biovision) per manufacturer's instructions (24).

HIV-1_BaL_ (25) was provided by the NIH AIDS Research & Reference Reagent Program (http://www.aidsreagent.org/). Viral stocks were by passaging through activated PBMCs for 11 days (26).

### Cell culture conditions

All cell cultures were maintained at 37 °C in an atmosphere containing 5% CO_2_. TZM-bl cells (27–29) were grown in Dulbecco's Minimal Essential Medium (DMEM) (Sigma-Aldrich, Inc., St. Louis, MO) containing 10% fetal calf serum (FCS), 2 mM L-glutamine and antibiotics (100 U of penicillin/ml, 100 μg of streptomycin/ml). PM-1 cells (30) (AIDS reagent project, National Institute for Biological Standards and Control, UK) were maintained in RPMI 1,640 medium containing 10% FBS, 2 mM L-glutamine and antibiotics (100 U of penicillin/ml and 100 μg of streptomycin/ml).

### Patients and tissue explants

Surgically-resected specimens of human ecto-cervical tissue were collected at St. Mary's Hospital, Imperial College Healthcare NHS Trust, London, UK. All tissues were collected after receiving signed informed consent from all patients through the Imperial College Healthcare Tissue Bank approved by Research Ethics Committee Wales (IRAS 17/WA/0161). All patients were HIV-negative. Mucosal tissue specimens were transported to the laboratory and processed less than 1 h after resection. Upon arrival in the laboratory, resected tissue was cut into 2–3 mm^3^ explants comprising epithelial and stromal layers as described previously (31). Non-polarized tissue explants were maintained with DMEM containing 10% fetal calf serum, 2 mM L-glutamine and antibiotics (100 U of penicillin/ml, 100 μg of streptomycin/ml, 80 μg of gentamicin/ml).

### Infectivity and inhibition assays

]The infectivity of virus preparations was estimated in TZM-bl cells (by luciferase quantitation of cell lysates, Promega, Madison, WI) and PBMCs (by measure of p24 antigen content in cell culture supernatant). The extent of luciferase expression was recorded in relative light units (r.l.u) as described previously (32). Viral p24 content in supernatant was measured with HIV-1 p24 ELISA (Innotest HIV antigen ELISA, Fujirebio Europe, Belgium) following manufacturer's instructions. Experiments were performed using a standardized amount of virus culture supernatant normalized for infectivity. Cells or tissue explants were incubated with serial dilutions of APs for 1 h at 37 °C. After 1 h at 37 °C, virus was added to TZM-bl cells (10^3.3^ TCID_50_/ml) and left for the time of the experiment (2 days). Alternatively, tissue explants were challenged with HIV-1_BaL_ at 10^4^ TCID_50_/ml. After 2 h of incubation, explants were washed with PBS, transferred to fresh plates and cultured for 24 h. Then, explants were once more transferred to fresh plates to harvest cells that might have migrated out of the tissue. Cervical migratory cells were either transferred to 96-well plates containing PM-1 cells or counted. Tissue explants and co-cultures with PM-1 cells were cultured for 15 days in the absence of inhibitor. Approximately 50% of supernatant was harvested every 3 to 4 days and replaced with fresh media. Infectivity was evaluated in supernatants by analysis of p24 concentration (Innotest HIV antigen ELISA).

### Viability assays

Viability in the presence of antiproteases was determined by measuring tetrazolium salt [3-(4,5-dimethyl-2-thiazolyl)-2,5-diphenyl-2H-tetrazolium bromide (MTT)] (Sigma-Aldrich, Inc., St. Louis, MO) cleavage into a blue product (formazan) by viable cells (33). Briefly, cells and explants were incubated or not with antiproteases or Nonoxydol-9 (N-9) for 24 h. Then, culture supernatants were removed and 0.5 mg/ml of MTT added. Plates were incubated for 3 h at 37 °C. MTT solution was aspirated and lysis buffer (98% Isopropanol with 2% HCL 2N) added for 30 min at 37 °C before measurement of optical density (O.D) with a Synergy-HT plate reader (BioTek, Winooski, VT). Alternatively, after incubating tissue explants with MTT solution, dry weight was recorded. Absorbance was measured after overnight incubation with methanol at room temperature. OD values were corrected for explant dry weight.

### Statistical and mathematical analysis

IC_50_ values were calculated from sigmoid curve fitted (Prism, GraphPad) fulfilling the criterion of R^2^ > 0.7. Statistical significance was determined using a two-tailed unpaired Student *t* test, and *P* ≤ 0.05 was considered statistically significant.

## Results

### Inhibitory activity of APs in TZM-bl cells

**Figure 1:**
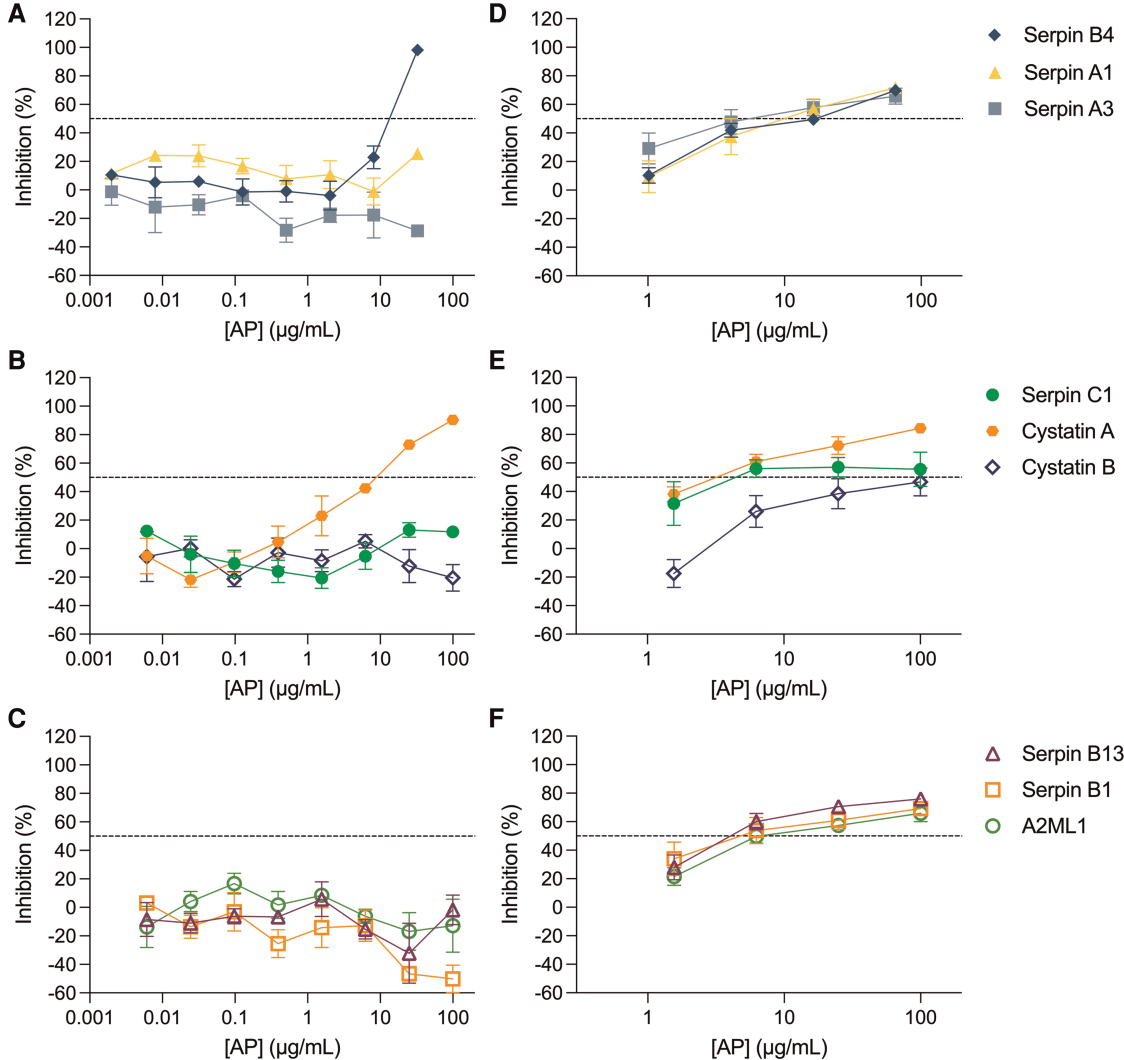
Activity of antiproteases in cellular and tissue explant models. Antiproteases (APs) were titrated against HIV-1_BaL_ in TZM-bl cells (**A–C**) and ecto-cervical explants (**D**–**F**). The percentage of inhibition by each AP was normalized relative to the r.l.u values obtained for cells or p24 values obtained for explants not exposed to virus (0% infectivity) and for cells or explants infected with virus in the absence of AP (100% infectivity). Data are means (± SEM) from at least two independent experiments performed in triplicate. Dashed line in each panel represents 50% of inhibition.

The inhibitory activity of the non-formulated APs, serpin B4, serpin A1, serpin A3, serpin C1, cystatin A, cystatin B, serpin B13, serpin B1 and A2ML1, was assessed in TZM-bl cells against the clade B R5-tropic isolate HIV-1_BaL_ (Figures 1A–C, Table 1). The estimated concentration of these antiproteases in cervical secretions is in the μg/ml range, hence the maximum concentrations tested were 100 μg/ml or 32.5 μg/ml depending on manufactured protein stock. In this cellular model, dose-response curves were only obtained for serpin B4 and cystatin A with IC_50_ values of 15.60 ± 2.57 μg/ml and 6.29 ± 2.98 μg/ml, respectively. No cytotoxicity was observed by MTT viability assay (Supplementary Figure S1A).

**Table 1:** IC_50_ of antiproteases against HIV-1_BaL_ in different models.

Antiprotease	IC_50_ (μg/ml)^a^	
	TZM-bl cells	Ecto-cervical explants
Serpin B4	15.73 ± 1.55	14.60 ± 8.36
Serpin A1	N/A	12.41 ± 9.23
Serpin A3	N/A	11.01 ± 9.51
Serpin C1	N/A	6.33 ± 2.73
Cystatin A	6.29 ± 1.72	12.72 ± 8.99
Cystatin B	N/A	N/A
Serpin B13	N/A	5.12 ± 1.05
Serpin B1	N/A	6.16 ± 4.45
A2ML1	N/A	10.42 ± 3.48

N/A: value could not be calculated within the range of concentrations tested.

^a^ Data are means (± SEM) derived from at least two independent experiments performed in triplicate.

### HIV-1 prophylaxis potential of APs in cervical tissue

The inhibitory profile measured in TZM-bl cells can only recapitulate a limited number of inhibitory mechanisms. Furthermore, considering the limited predictive power of TZM-bl cells for mucosal compartments and to evaluate the potential of mucosal proteins as a pre-exposure prophylaxis strategy, we tested the nine APs in a more relevant *ex vivo* model of human ecto-cervical tissue. Titration of the APs in ecto-cervical tissue explants revealed dose-response curves against HIV-1_BaL_ (Figures 1D–F) with different inhibitory profiles. For all APs, except cystatin B, an IC_50_ value could be calculated (Table 1); however, cystatin A was the only protein reaching inhibitory levels above 80% within the range of concentrations tested. None of the APs were cytotoxic in cervical explants withing the range of concentrations tested (Supplementary Figure S1B).

**Figure 2:**
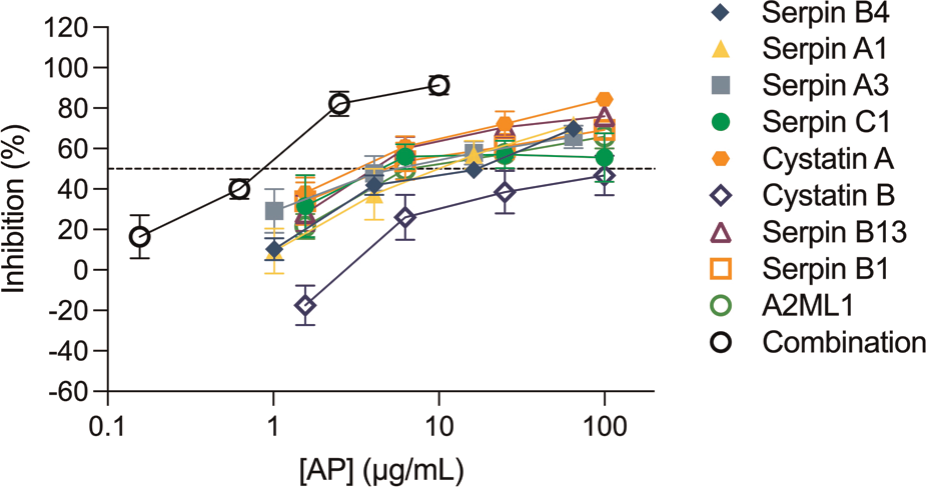
Combination of nine antiproteases in ecto-cervical explants are more active against HIV-1_BaL_ than each individual drugs. The dose-response curve of each antiprotease (AP) was compared with than of the combination of the nine APs. The percentage of inhibition by each AP and by the combination was normalized relative to the p24 values obtained for ecto-cervical explants not exposed to virus (0% infectivity) and for explants infected with virus in the absence of APs (100% infectivity). Data are means (± SEM) from at least two independent experiments performed in triplicate. Dashed line represents 50% of inhibition.

We next considered the potential of a combinatorial approach with all nine APs mimicking their presence in the cervicovaginal tract. When all nine APs were combined at the same concentration and titrated maintaining the same proportion, a higher inhibition level was reached in ecto-cervical explants with the nine AP-combination than with each of the APs titrated individually (Figure 2). The dose-response curve showed a reduction in the IC_50_, with a value of 0.79 ± 0.039 μg/ml for the nine APs combination (reaching significance towards the IC_50_ of serpin C1 *P *= 0.0284, cystatin B *P *= 0.0054 and serpin B13 *P *= 0.0498 when tested alone) and an increase of the higher maximum level of inhibition of 91.26 ± 1.824% reached within the range of concentrations tested.

### APs are associated with decreased count of *ex vivo* ecto-cervical migratory cells

**Figure 3:**
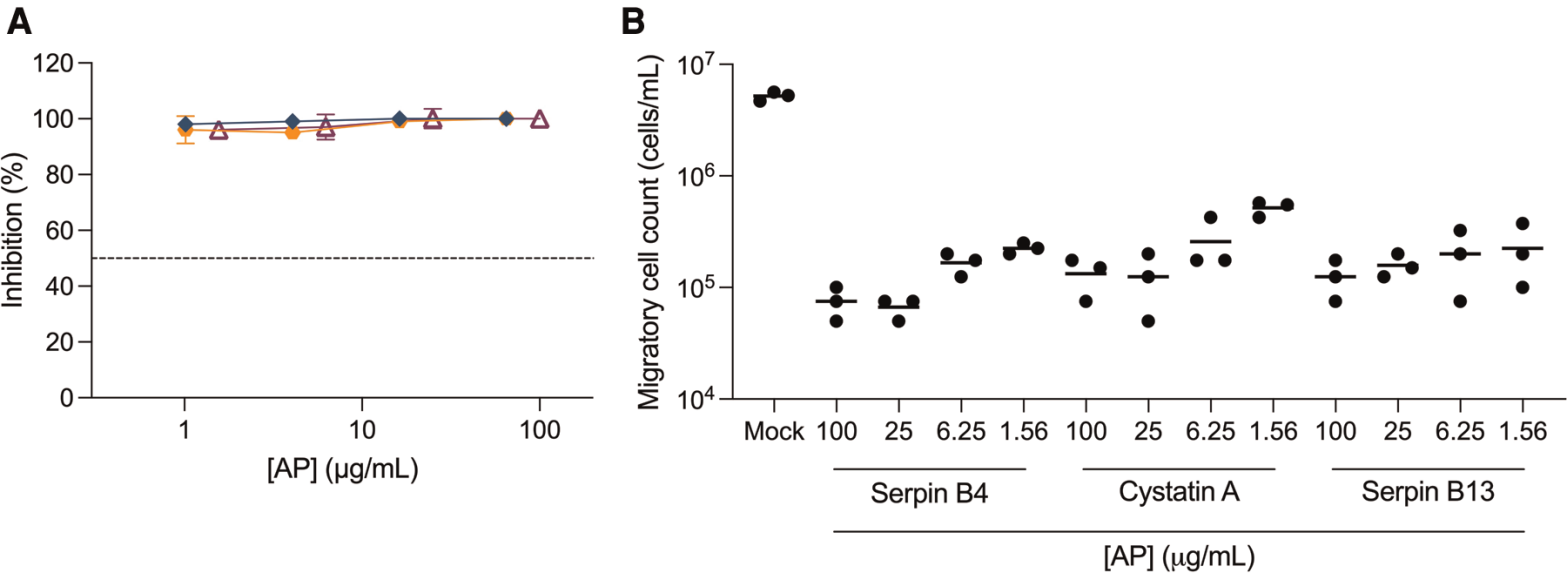
**Inhibition of** ***trans-*****infection by antiproteases.** Ecto-cervical explants were treated with antiproteases (APs), serpin B4 (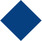), cystatin A (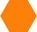), serpin B13 (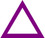), prior to viral challenge and after four washes with PBS, transferred to new plates. Migratory cells were harvested after 24 h of culture and were either (**A**) transferred to 96-well plates containing PM-1 cells or (**B**) counted in a total volume of 200 μL. Co-cultures with PM-1 cells were kept for 15 days. The concentrations of p24 in the harvested supernatants were quantified by ELISA and the extent of inhibition calculated. The percentage of inhibition was normalized relative to the p24 values obtained for cultures not exposed to virus (0% infectivity) and for cultures infected with virus in the absence of compound (100% infectivity). Dashed line represents 50% of inhibition. Data are means (± SEM) from three independent experiments performed in triplicate.

To further assess the potential of APs as PrEP candidates, we measured their inhibitory activity against *trans-*infection between cervical migratory cells and CD4^+^ T cells in a co-culture model of migratory cells isolated from ecto-cervical explants and a CD4^+^ T cell line, PM-1 cells. We initially evaluated serpin B4, cystatin A and serpin B13. Surprisingly, no viral replication was observed in culture supernatants (Figure 3A). To investigate this non titratable inhibition, new ecto-cervical explants from independent donors were dosed with these APs. A significant reduction in migratory cell count was measured in treated explants compared to the number of migratory cells measured in control explant cultures not dosed with APs (*P *< 0.0001 for all conditions) (Figure 3B).

## Discussion

We have evaluated, side by side, the activity of nine mucosal APs, including serpins and cystatins, in cellular and tissue models of *cis-* and *trans-*infection. Cystatin A was the most potent protein candidate in all models used; however, the order of inhibitory potency for the nine APs was different in each model. The maximum inhibitory level achieved by the most potent AP in TZM-bl cells, serpin B4, was not reached in ecto-cervical tissue explants. These discrepancies highlight the importance of pre-clinical evaluation with models that mimic the mucosal environment. Furthermore, analysis of the inhibitory capacity of serpin B4, serpin B13 and cystatin A against *trans-*infection revealed another potential anti-viral mechanism of protection for these proteins, with a significant reduction in the number of cells migrating out of ecto-cervical explants after overnight culture post-*ex vivo* dosing with APs. Cervical migratory cells are dendritic cells (DCs) (31), and decreased migration of cells from the ecto-cervix prevents onward dissemination of HIV to secondary lymphoid tissue (34). No cytotoxicity was observed in ecto-cervical explants following exposure to any of the AP tested, hence the reduction in migratory cell count is not due to a cytotoxic effect. This is in line with reports linking certain antiproteases with anti-apoptotic functions (35). Migration of cervical DCs, including myeloid DCS, plasmacytoid DCs and Langerhans cells, has been shown to be modulated by pro-inflammatory cytokines such as TNF-α, IL-1β, IL-8 and MIP-1β using ecto-cervical tissue explants cultures (36). Cystatin A has been shown to inhibit IL-8 production by keratinocytes (37). Serpin B13 inhibits cathepsin K, L and V. Cathepsin K has been shown to induce secretion of the pro-inflammatory cytokine IL-6 (38) and to facilitate immune cell migration with cathepsin L (39). Furthermore, the later has been shown to induce proliferation of CD4^+^ T cells (40). Hence, inhibition of these cathepsins by serpin B13 could reduce the number of HIV target cells and the levels of IL-6, which is known to be upregulated during the acute phase of HIV infection in the female genital tract (41). However, increased expression of serpin B4 and cystatin A have been described in chronic inflammatory conditions of the skin with infiltration of dendritic cells, macrophages, Th1 cells and neutrophils (42, 43). Hence, further investigation is necessary to assess the impact of these protease inhibitory proteins on the mucosal environment and immunology of the female genital tract.

The APs included in this study were found to be overabundant in the HIV-resistant women from the Punwami Sex Worker cohort, however it remains unclear what triggered this altered mucosal expression levels. Recent studies have shown that serpin and cystatin levels in the female genital tract can be modulated by hormonal treatment (44, 45) or even by chronic sexual abuse (46). The initial combinatorial study performed in ecto-cervical explants aims at reproducing this increased expression of not just one, but all the antiproteases identified in this cohort. The distinct inhibitory potency observed in the TZM-bl cell and in the ecto-cervical explant cultures indicates that the mechanism of action is linked to the anti-inflammatory response induced by these proteins more than by a direct anti-viral mechanism. To our knowledge no study has evaluated the effect of the combination of these anti-proteases on pro-inflammatory cytokines/chemokines and other mucosal factors that could affect the susceptibility to HIV-1 infection.

The tissue explant model is increasingly being used as a pre-clinical tool to reduce the late-stage failure of HIV prevention candidates (47) and in early clinical trials (48–53). Furthermore, a multi-site study has shown that protocol standardization provides measurement consistency among different laboratories (54). This model recapitulates the histological and immunological characteristics of the genital mucosae and early responses to stimuli can be measured (55, 56). However, limitations include (i) progressive loss of architecture despite the maintenance of CD4:CD8 T cell ratios and sufficient viability to sustain viral replication for more than 10 days (57); (ii) paucity of data regarding preservation of immune competence (58); (iii) limitation to demonstrate sterilizing protection.

To assess the combinatorial activity (synergy/additivity/antagonism) of anti-viral candidates, the Chou-Talalay equation (59) has often been used. However, to apply this equation correctly, the slopes of all the dose-response curves compared must be parallel and the activity of the candidate must cover the full range between 0% and 100% of inhibition. However, donor-to-donor variation of the explant model, assessment of molecules with different mechanisms of action and limited potency for some, makes this impossible to achieve. Hence, we provided the IC_50_ value to show the reduction in this value and the maximum inhibitory potency achieved within the range of concentration tested as indicators of increased anti-viral activity.

Our study has several limitations, including the sparse number of explants that can be cut from each ecto-cervical specimen limiting the breath of the titration and the number of proteins that can be compared within a same donor. The 80%–85% purity of the APs is due to the presence of deletion peptide sequences generated during synthesis and which could affect the anti-viral potency of these proteins or reproducibility of assays. Hence, peptide candidates of higher purity could provide greater inhibitory potency. No analysis of mucosal cytokine/chemokine profile linked to inflammatory responses to APs was performed in this initial study. Furthermore, inhibitory activity was only assessed against a laboratory-adapted clade B virus and not against transmitted founder isolates from various clades. In future studies, it will also be important to assess the potential presence of HIV genetic material in the migratory cells to better define the mechanism of *trans-*infection inhibition.

The encouraging results obtained in this pilot study support further investigation to assess the mechanism of action of these proteins in the mucosal environment, with a focus on the potential modulation of inflammatory responses. Furthermore, it will be important the define the impact of such modulation on the migratory capacity of cervical DCs and, therefore, on the inhibition of the local expansion and viral dissemination to draining lymph nodes that occur following establishment of the initial founder population during mucosal HIV-1 transmission (34). Evaluation of their activity with increased dosing regimens will inform formulation strategies such as sustained delivery. Understanding the mechanism of action and pharmacological profile will be important for the dosing and formulation strategy. Furthermore, these host mucosal proteins will now be evaluated in combination with ARVs and against multiple viral clades and ARV-resistant isolates. Hence, this study constitutes the base for further development of host mucosal proteins as HIV PrEP candidates.

## Data Availability

The raw data supporting the conclusions of this article will be made available by the authors, without undue reservation.

## References

[1] WHO. Focus on key populations in national HIV strategic plans in the african region. Congo: Regional Office for Africa (2018).

[2] Advocacy CaG. UNAIDS (2021). Available at: https://www.unaids.org/sites/default/files/media_asset/UNAIDS_FactSheet_en.pdf

[3] Pennings PS. HIV drug resistance: problems and perspectives. Infect Dis Rep. (2013) 5(Suppl 1):e5. 10.4081/idr.2013.s1.e5 24470969 PMC3892620

[4] Snedecor SJ Sudharshan L Nedrow K Bhanegaonkar A Simpson KN Haider S Burden of nonnucleoside reverse transcriptase inhibitor resistance in HIV-1-infected patients: a systematic review and meta-analysis. AIDS Res Hum Retroviruses. (2014) 30(8):753–68. 10.1089/aid.2013.0262 24925216 PMC4118702

[5] Fowke KR Nagelkerke NJ Kimani J Simonsen JN Anzala AO Bwayo JJ Resistance to HIV-1 infection among persistently seronegative prostitutes in Nairobi, Kenya. Lancet. (1996) 348(9038):1347–51. 10.1016/S0140-6736(95)12269-2 8918278

[6] Simonsen JN Plummer FA Ngugi EN Black C Kreiss JK Gakinya MN HIV Infection among lower socioeconomic strata prostitutes in Nairobi. AIDS. (1990) 4(2):139–44. 10.1097/00002030-199002000-00007 2328096

[7] Burgener A Boutilier J Wachihi C Kimani J Carpenter M Westmacott G Identification of differentially expressed proteins in the cervical mucosa of HIV-1-resistant sex workers. J Proteome Res. (2008) 7(10):4446–54. 10.1021/pr800406r 18707157

[8] Burgener A Rahman S Ahmad R Lajoie J Ramdahin S Mesa C Comprehensive proteomic study identifies serpin and cystatin antiproteases as novel correlates of HIV-1 resistance in the cervicovaginal mucosa of female sex workers. J Proteome Res. (2011) 10(11):5139–49. 10.1021/pr200596r 21973077

[9] Law RH Zhang Q McGowan S Buckle AM Silverman GA Wong W An overview of the serpin superfamily. Genome Biol. (2006) 7(5):216. 10.1186/gb-2006-7-5-216 16737556 PMC1779521

[10] Askew DJ Silverman GA. Intracellular and extracellular serpins modulate lung disease. J Perinatol. (2008) 28(Suppl 3):S127–35. 10.1038/jp.2008.150 19057604 PMC7104463

[11] Wilson TJ Nannuru KC Singh RK. Cathepsin G recruits osteoclast precursors via proteolytic activation of protease-activated receptor-1. Cancer Res. (2009) 69(7):3188–95. 10.1158/0008-5472.CAN-08-1956 19293192

[12] Sambrano GR Huang W Faruqi T Mahrus S Craik C Coughlin SR. Cathepsin G activates protease-activated receptor-4 in human platelets. J Biol Chem. (2000) 275(10):6819–23. 10.1074/jbc.275.10.6819 10702240

[13] Chertov O Ueda H Xu LL Tani K Murphy WJ Wang JM Identification of human neutrophil-derived cathepsin G and azurocidin/CAP37 as chemoattractants for mononuclear cells and neutrophils. J Exp Med. (1997) 186(5):739–47. 10.1084/jem.186.5.739 9271589 PMC2199011

[14] Hashemi FB Mollenhauer J Madsen LD Sha BE Nacken W Moyer MB Myeloid-related protein (MRP)-8 from cervico-vaginal secretions activates HIV replication. AIDS. (2001) 15(4):441–9. 10.1097/00002030-200103090-00002 11242140

[15] Herrick S Ashcroft G Ireland G Horan M McCollum C Ferguson M. Up-regulation of elastase in acute wounds of healthy aged humans and chronic venous leg ulcers are associated with matrix degradation. Lab Invest. (1997) 77(3):281–8. PMID: 9314951 9314951

[16] Ashcroft GS Greenwell-Wild T Horan MA Wahl SM Ferguson MW. Topical estrogen accelerates cutaneous wound healing in aged humans associated with an altered inflammatory response. Am J Pathol. (1999) 155(4):1137–46. 10.1016/S0002-9440(10)65217-0 10514397 PMC1867002

[17] Ashcroft GS Lei K Jin W Longenecker G Kulkarni AB Greenwell-Wild T Secretory leukocyte protease inhibitor mediates non-redundant functions necessary for Normal wound healing. Nat Med. (2000) 6(10):1147–53. 10.1038/80489 11017147

[18] Munch J Standker L Adermann K Schulz A Schindler M Chinnadurai R Discovery and optimization of a natural HIV-1 entry inhibitor targeting the gp41 fusion peptide. Cell. (2007) 129(2):263–75. 10.1016/j.cell.2007.02.042 17448989

[19] Pott GB Chan ED Dinarello CA Shapiro L. Alpha-1-antitrypsin is an endogenous inhibitor of proinflammatory cytokine production in whole blood. J Leukoc Biol. (2009) 85(5):886–95. 10.1189/jlb.0208145 19197072 PMC2669404

[20] Shapiro L Pott GB Ralston AH. Alpha-1-antitrypsin inhibits human immunodeficiency virus type 1. Faseb J. (2001) 15(1):115–22. 10.1096/fj.00-0311com 11149899

[21] Bryan CL Beard KS Pott GB Rahkola J Gardner EM Janoff EN HIV Infection is associated with reduced serum alpha-1-antitrypsin concentrations. Clin Invest Med. (2010) 33(6):E384–9. 10.25011/cim.v33i6.14589 21134340

[22] Kramer HB Lavender KJ Qin L Stacey AR Liu MK di Gleria K Elevation of intact and proteolytic fragments of acute phase proteins constitutes the earliest systemic antiviral response in HIV-1 infection. PLoS Pathog. (2010) 6(5):e1000893. 10.1371/journal.ppat.1000893 20463814 PMC2865525

[23] Zhou X Shapiro L Fellingham G Willardson BM Burton GF. HIV Replication in CD4+ T lymphocytes in the presence and absence of follicular dendritic cells: inhibition of replication mediated by alpha-1-antitrypsin through altered IkappaBalpha ubiquitination. J Immunol. (2011) 186(5):3148–55. 10.4049/jimmunol.1001358 21263074 PMC3101708

[24] Aboud L. Defining the HIV neutralizing activity of antiproteases within the female genital tract and evaluating the HIV inhibitory mechanism of Serpin B1 [Doctoral thesis]. Winnipeg, Manitoba, Canada: University of Manitoba (2016).

[25] Gartner S Markovits P Markovitz DM Kaplan MH Gallo RC Popovic M. The role of mononuclear phagocytes in HTLV-III/LAV infection. Science. (1986) 233(4760):215–9. 10.1126/science.3014648 3014648

[26] Gordon CJ Muesing MA Proudfoot AE Power CA Moore JP Trkola A. Enhancement of human immunodeficiency virus type 1 infection by the CC-chemokine RANTES is independent of the mechanism of virus-cell fusion. J Virol. (1999) 73(1):684–94. 10.1128/JVI.73.1.684-694.1999 9847374 PMC103875

[27] Derdeyn CA Decker JM Sfakianos JN Wu X O'Brien WA Ratner L Sensitivity of human immunodeficiency virus type 1 to the fusion inhibitor T-20 is modulated by coreceptor specificity defined by the V3 loop of gp120. J Virol. (2000) 74(18):8358–67. 10.1128/JVI.74.18.8358-8367.2000 10954535 PMC116346

[28] Platt EJ Wehrly K Kuhmann SE Chesebro B Kabat D. Effects of CCR5 and CD4 cell surface concentrations on infections by macrophagetropic isolates of human immunodeficiency virus type 1. J Virol. (1998) 72(4):2855–64. 10.1128/JVI.72.4.2855-2864.1998 9525605 PMC109730

[29] Wei X Decker JM Liu H Zhang Z Arani RB Kilby JM Emergence of resistant human immunodeficiency virus type 1 in patients receiving fusion inhibitor (T-20) monotherapy. Antimicrob Agents Chemother. (2002) 46(6):1896–905. 10.1128/AAC.46.6.1896-1905.2002 12019106 PMC127242

[30] Lusso P Cocchi F Balotta C Markham PD Louie A Farci P Growth of macrophage-tropic and primary human immunodeficiency virus type 1 (HIV-1) isolates in a unique CD4+ T-cell clone (PM1): failure to downregulate CD4 and to interfere with cell-line-tropic HIV-1. J Virol. (1995) 69(6):3712–20. 10.1128/jvi.69.6.3712-3720.1995 7745720 PMC189087

[31] Hu Q Frank I Williams V Santos JJ Watts P Griffin GE Blockade of attachment and fusion receptors inhibits HIV-1 infection of human cervical tissue. J Exp Med. (2004) 199(8):1065–75. 10.1084/jem.20022212 15078900 PMC2211899

[32] Herrera C Cranage M McGowan I Anton P Shattock RJ. Reverse transcriptase inhibitors as potential colorectal microbicides. Antimicrob Agents Chemother. (2009) 53(5):1797–807. 10.1128/AAC.01096-08 19258271 PMC2681527

[33] Slater TF Sawyer B Straeuli U. Studies on succinate-tetrazolium reductase systems. Iii. Points of coupling of four different tetrazolium salts. Biochim Biophys Acta. (1963) 77:383–93. 10.1016/0006-3002(63)90513-4 14089413

[34] Li Q Estes JD Schlievert PM Duan L Brosnahan AJ Southern PJ Glycerol monolaurate prevents mucosal SIV transmission. Nature. (2009) 458(7241):1034–8. 10.1038/nature07831 19262509 PMC2785041

[35] Bots M Medema JP. Serpins in T cell immunity. J Leukoc Biol. (2008) 84(5):1238–47. 10.1189/jlb.0208140 18641264

[36] Shey MS Maharaj N Archary D Ngcapu S Garrett N Abdool Karim S Modulation of female genital tract-derived dendritic cell migration and activation in response to inflammatory cytokines and toll-like receptor agonists. PloS one. (2016) 11(5):e0155668. 10.1371/journal.pone.0155668 27171482 PMC4865202

[37] Kato T Takai T Mitsuishi K Okumura K Ogawa H. Cystatin A inhibits IL-8 production by keratinocytes stimulated with der p 1 and der f 1: biochemical skin barrier against mite cysteine proteases. J Allergy Clin Immunol. (2005) 116(1):169–76. 10.1016/j.jaci.2005.03.044 15990791

[38] Muller S Faulhaber A Sieber C Pfeifer D Hochberg T Gansz M The endolysosomal cysteine cathepsins L and K are involved in macrophage-mediated clearance of Staphylococcus aureus and the concomitant cytokine induction. FASEB J. (2014) 28(1):162–75. 10.1096/fj.13-232272 24036885

[39] Fonovic M Turk B. Cysteine cathepsins and extracellular matrix degradation. Biochim Biophys Acta. (2014) 1840(8):2560–70. 10.1016/j.bbagen.2014.03.017 24680817

[40] Liszewski MK Kolev M Le Friec G Leung M Bertram PG Fara AF Intracellular complement activation sustains T cell homeostasis and mediates effector differentiation. Immunity. (2013) 39(6):1143–57. 10.1016/j.immuni.2013.10.018 24315997 PMC3865363

[41] Bebell LM Passmore JA Williamson C Mlisana K Iriogbe I van Loggerenberg F Relationship between levels of inflammatory cytokines in the genital tract and CD4+cell counts in women with acute HIV-1 infection. J Infect Dis. (2008) 198(5):710–4. 10.1086/590503 18643751

[42] Sun Y Sheshadri N Zong WX. SERPINB3 And B4: from biochemistry to biology. Semin Cell Dev Biol. (2017) 62:170–7. 10.1016/j.semcdb.2016.09.005 27637160 PMC5318283

[43] Magister S Kos J. Cystatins in immune system. J Cancer. (2013) 4(1):45–56. 10.7150/jca.5044 23386904 PMC3564246

[44] Biswas S Chen E Gao Y Lee S Hewlett I Devadas K. Modulation of HIV replication in monocyte-derived macrophages (MDM) by host antiviral factors secretory leukocyte protease inhibitor and serpin family C member 1 induced by steroid hormones. Viruses. (2022) 14(1):95. 10.3390/v14010095 35062299 PMC8777669

[45] Bradley F Franzen Boger M Kaldhusdal V Ahlberg A Edfeldt G Lajoie J Multi-omics analysis of the cervical epithelial integrity of women using depot medroxyprogesterone acetate. PLoS Pathog. (2022) 18(5):e1010494. 10.1371/journal.ppat.1010494 35533147 PMC9119532

[46] Ghosh M Daniels J Pyra M Juzumaite M Jais M Murphy K Impact of chronic sexual abuse and depression on inflammation and wound healing in the female reproductive tract of HIV-uninfected and HIV-infected women. PloS one. (2018) 13(6):e0198412. 10.1371/journal.pone.0198412 29894487 PMC5997353

[47] Herrera C Shattock RJ. Candidate microbicides and their mechanisms of action. Curr Top Microbiol Immunol. (2014) 383:1–25. 10.1007/82_2013_326 23612992

[48] Anton PA Cranston RD Kashuba A Hendrix CW Bumpus NN Richardson-Harman N RMP-02/MTN-006: a phase 1 rectal safety, acceptability, pharmacokinetic, and pharmacodynamic study of tenofovir 1% gel compared with oral tenofovir disoproxil fumarate. AIDS Res Hum Retroviruses. (2012) 28(11):1412–21. 10.1089/aid.2012.0262 22943559 PMC3484811

[49] Fox J Tiraboschi JM Herrera C Else L Egan D Dickinson L Brief report: pharmacokinetic/pharmacodynamic investigation of single-dose oral maraviroc in the context of HIV-1 Pre-exposure prophylaxis. J Acquir Immune Defic Syndr. (2016) 73(3):252–7. 10.1097/QAI.0000000000001108 27727157

[50] Herrera C Lwanga J Lee M Mantori S Amara A Else L Pharmacokinetic/pharmacodynamic investigation of raltegravir with or without lamivudine in the context of HIV-1 pre-exposure prophylaxis (PrEP). J Antimicrob Chemother. (2021) 76(8):2129–36. 10.1093/jac/dkab136 33993302 PMC8325523

[51] McGowan I Cranston RD Duffill K Siegel A Engstrom JC Nikiforov A A phase 1 randomized, open label, rectal safety, acceptability, pharmacokinetic, and pharmacodynamic study of three formulations of tenofovir 1% gel (the CHARM-01 study). PLoS One. (2015) 10(5):e0125363. 10.1371/journal.pone.0125363 25942472 PMC4420274

[52] Richardson-Harman N Hendrix CW Bumpus NN Mauck C Cranston RD Yang K Correlation between compartmental tenofovir concentrations and an ex vivo rectal biopsy model of tissue infectibility in the RMP-02/MTN-006 phase 1 study. PLoS One. (2014) 9(10):e111507. 10.1371/journal.pone.0111507 25350130 PMC4211741

[53] Richardson-Harman N Mauck C McGowan I Anton P. Dose-response relationship between tissue concentrations of UC781 and explant infectibility with HIV type 1 in the RMP-01 rectal safety study. AIDS Res Hum Retroviruses. (2012) 28(11):1422–33. 10.1089/aid.2012.0073 22900504 PMC3484735

[54] Richardson-Harman N Lackman-Smith C Fletcher PS Anton PA Bremer JW Dezzutti CS Multisite comparison of anti-human immunodeficiency virus microbicide activity in explant assays using a novel endpoint analysis. J Clin Microbiol. (2009) 47(11):3530–9. 10.1128/JCM.00673-09 19726602 PMC2772583

[55] Herrera C McRaven MD Laing KG Dennis J Hope TJ Shattock RJ. Early colorectal responses to HIV-1 and modulation by antiretroviral drugs. Vaccines. (2021) 9(3):231. 10.3390/vaccines9030231 33800213 PMC8000905

[56] Herrera C Veazey R Lemke MM Arnold K Kim JH Shattock RJ. Ex vivo evaluation of mucosal responses to vaccination with ALVAC and AIDSVAX of non-human primates. Vaccines. (2022) 10(2):187. 10.3390/vaccines10020187 35214645 PMC8879115

[57] Fletcher PS Elliott J Grivel JC Margolis L Anton P McGowan I Ex vivo culture of human colorectal tissue for the evaluation of candidate microbicides. Aids. (2006) 20(9):1237–45. 10.1097/01.aids.0000232230.96134.80 16816551

[58] Grivel JC Margolis L. Use of human tissue explants to study human infectious agents. Nat Protoc. (2009) 4(2):256–69. 10.1038/nprot.2008.245 19197269 PMC3427853

[59] Chou T-C Talalay P. A simple generalized equation for the analysis of multiple inhibitions of Michaelis-menten kinetic systems. J Biol Chem. (1977) 252(18):6438–42. 10.1016/S0021-9258(17)39978-7 893418

