# Supplementary material for: Pre-clinical evaluation of antiproteases as potential candidates for HIV-1 pre-exposure prophylaxis
Source: Front Reprod Health. 2022 Nov 21;4:998913. doi: 10.3389/frph.2022.998913 (PMC9720403; doi:10.3389/frph.2022.998913)
Supplement: Supplementary file 1 [file Image1.pdf]

Supplementary Material

1 Supplementary Figures and Tables

1.1 Supplementary Figure1

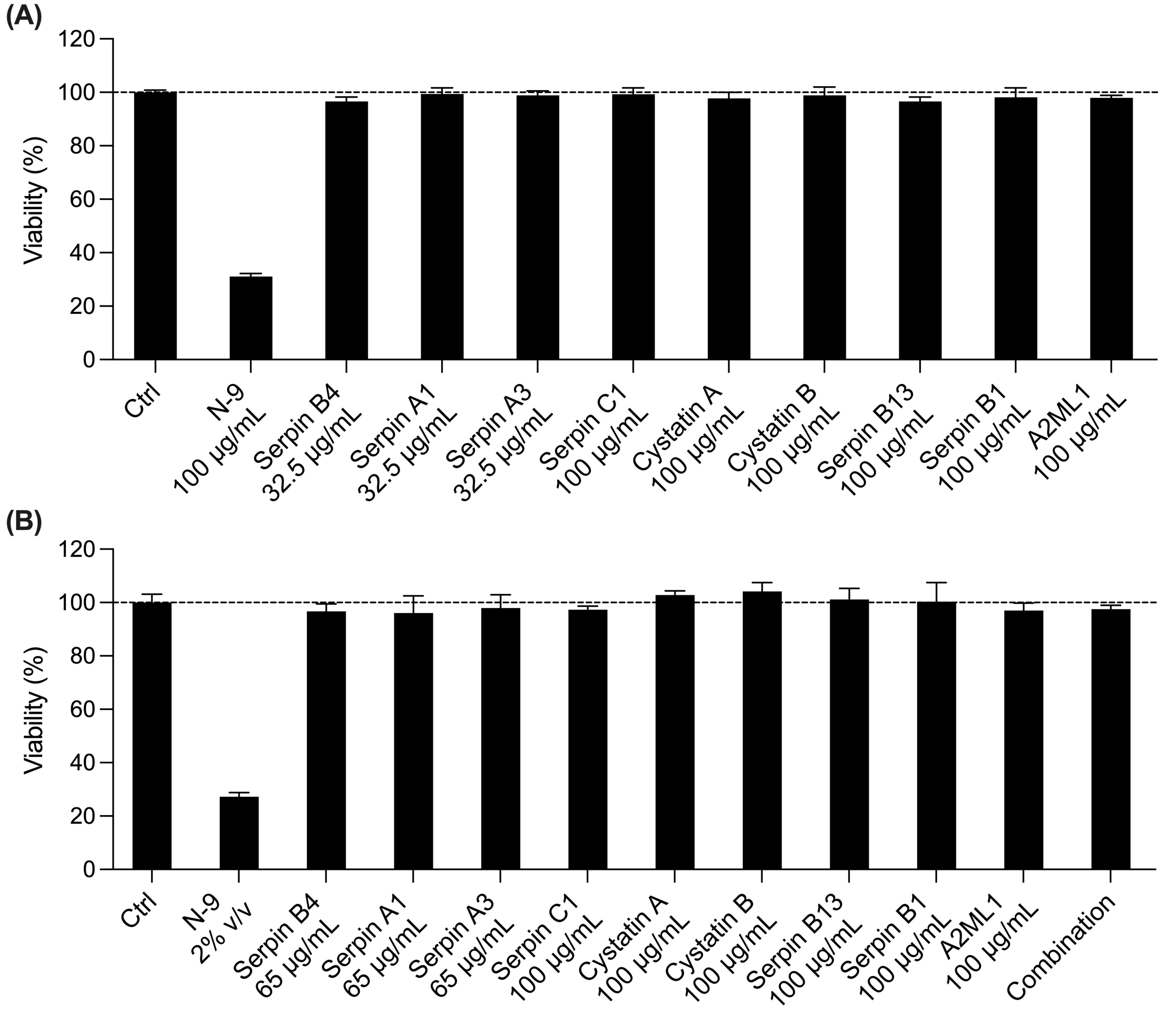

Supplementary Figure 1. Toxicity of antiproteases in cellular and tissue explant models. (A) TZM-bl cells and (B) ecto-cervical tissue explants were incubated or not with antiproteases or Nonoxydol-9 (N-9) for 24 h. Viability was assessed via MTT assay and percentage of viability was normalized relative to the OD values obtained for cells or explants not treated (100% viability) and for cells or explants treated with N-9 (0% viability). Data are means (± SEM) from one experiment performed in duplicate. Combination: combination of all antiproteases at the same concentration used when tested individually.
